# Supplementary material for: Core architecture of a bacterial type II secretion system
Source: Nat Commun. 2019 Nov 28;10:5437. doi: 10.1038/s41467-019-13301-3 (PMC6882859; doi:10.1038/s41467-019-13301-3)
Supplement: Supplementary file 1 — Supplementary Information [file 41467_2019_13301_MOESM1_ESM.pdf]

Supplementary Figures

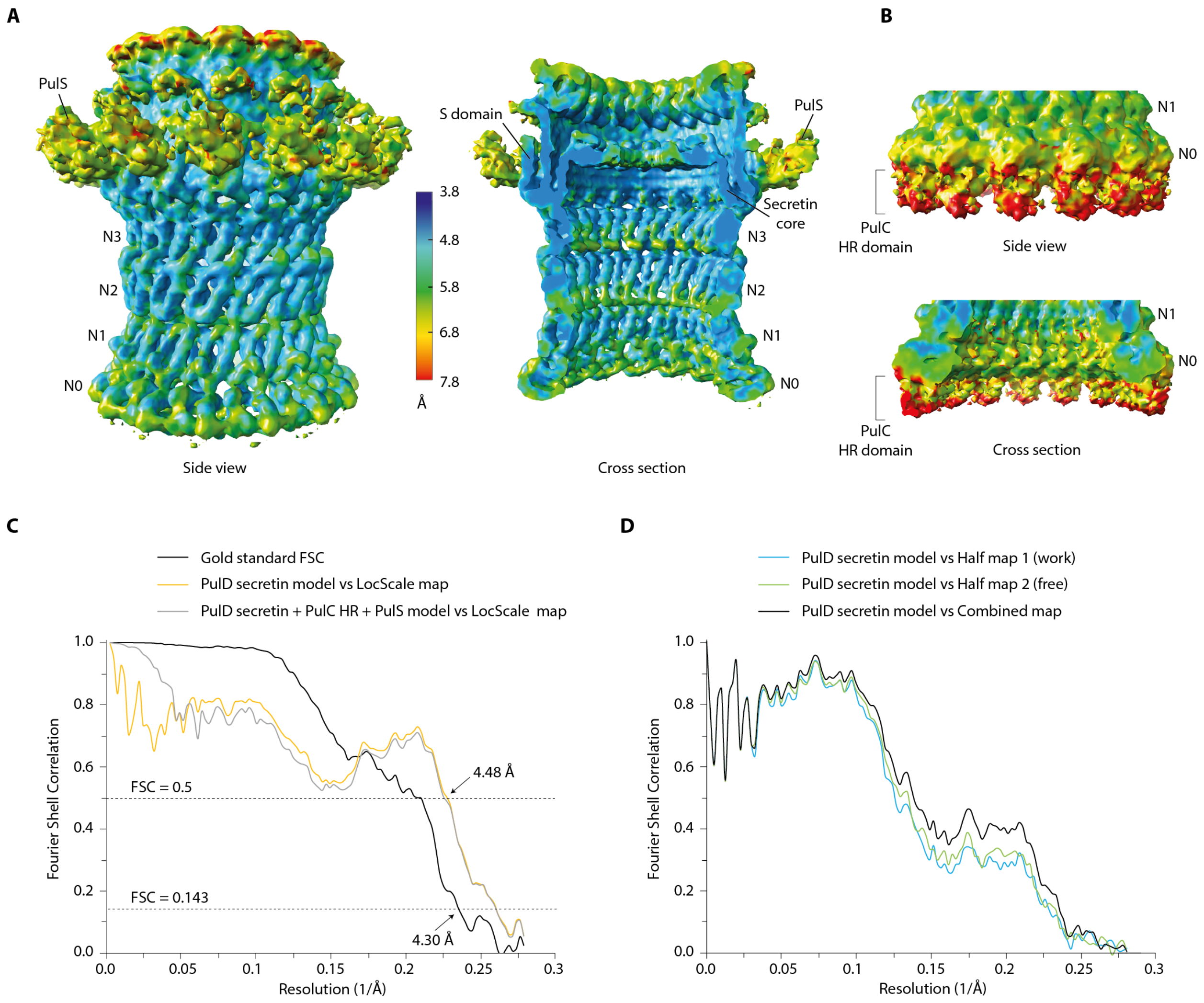

Figure S1. Outer membrane complex (OMC) local resolution map and FSC curves. (A) Unsharpened OMC map showing local resolution estimates calculated using ResMap[1] and contoured at 4.5σ. **(B)** Equivalent to **A** but contoured at 2σ so as to view the PulC HR domain densities at the secretin base. **(C)** Gold standard FSC curve of the OMC (black). FSC curves between the PulD secretin model and the LocScale[2] sharpened map (orange). FSC curves between the PulD secretin model with homology models of PulS and PulC HR domain fitted, and the LocScale sharpened map (grey). **(D)** Cross-validation for the PulD secretin model. PulD secretin model versus half map 1 (work- light blue), half map 2 (free- green), and combined (black). See Methods for further detail.

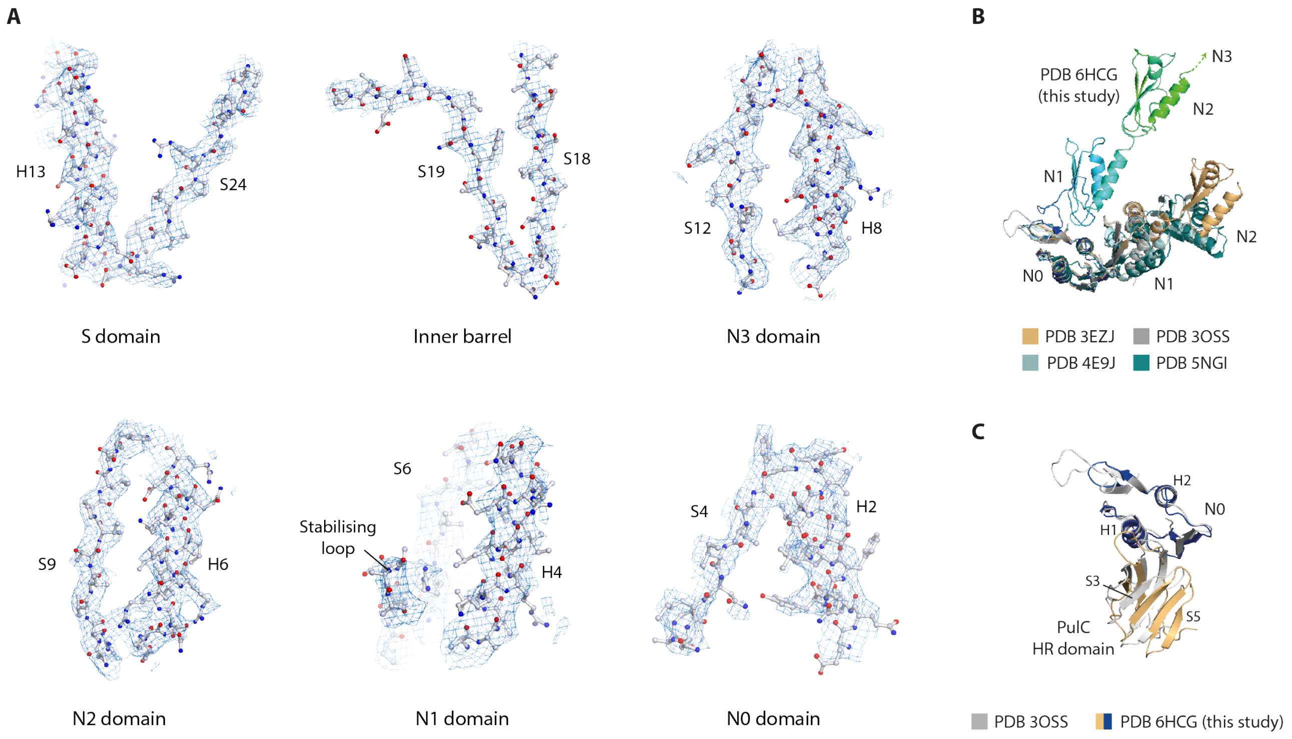

Figure S2. Map quality and PulS fit. (A) Selected regions of the OMC EM density map showing PulD secretin side chain detail and build. The map was sharpened with B factor = -142 Å^2 and contoured between 5-8σ. **(B)** Superposition of PulD N0, N1 and N2 domains with equivalent domains derived from various crystal structures including enterotoxigenic *E. coli* PDB 3EZJ and PDB 3OSS and *P. aeruginosa* PDB 4E9J and PDB 5NGI. **(C)** Superposition of PulD N0 domain in complex with PulC HR domain against the equivalent domains in the enterotoxigenic *E. coli* crystal structure PDB 3OSS. The complexes differ by RMSD Cα = 1.4 Å.

```
PA          -MSQPLLRALFAPSS------RSYVPAVLLSLALGIQAAHAENSGGNAFVPAGNQQEAHW   53
EC_K12      -------MKGLNKIT---------CCL--LA---------------ALLMPCAGHAENEQY   28
KP          -------MKSLRKML---------PALLILT---------------PLL---FSPAAAEEF   27
KO          ----MIIANVIRPFS---------LMLLVFA---------------ALL---FKPAAAEEF   30
EC_H10407   MFWRDITLSVWRKKTTGLKTKKRLLPLVLA---------------AALCSSPVWAEEATF   45
VC          -------MKYWLKKSSW-----------LLA---------------GSLLSTP-LAMANEF   27
                                        :                              :

            N0 domain
            S1   H1   S2   S3   H2
PA          TINLKDADIREFIDQISEITGETFVVDPRVKGQVSVVSKAQLSLSEVYQLFLSVMSTHGF   113
EC_K12      GANFNNADIRQFVEIVGQHLGKTILIDPSVQGTISVRSNDTFSQQEYYQFFLSILDLYGY   88
KP          SASFKGTDIQEFINTVSKNLNKTVIIDPSVRGTITVRSYDMLNEEQYYQFFLSVLDVYGF   87
KO          SASFKGTDIQEFINTVSKNLNKTVIIDPSVRGTITVRSYDMLNEEQYYQFFLSVLDVYGF   90
EC_H10407   TANFKDTDLKSFIETVGANLNKTIIMGPGVQGKVSIRTMTPLNERQYYQLFLNLLEAQGY   105
VC          SASFKGTDIQEFINIVGRNLEKTIIVDPSVRGKVDVRSFDTLNEEQYYSFFLSVLEVYGF   87
              .::.:*::.*:: :.    :*.::.* *:* : : :   :.  : *.:**.::.  *:

            N0 domain                                  N1 domain
            S4   S5   S6   H3
PA          TVVAQGDQ-ARIVPNAEAKTEA------GGGQSAPDRLETRVIQVQQSPVSELIPLIRPL   166
EC_K12      SVITLDNGFLKVVRSANVKTSPGMIAD-SSRPGVGDELVTRIVPLENVPARDLAPLLRQM   147
KP          AVINMNNGVLKVVRAKDAKTSAVPVAS-AAAPGEGDEVVTRVVPLTNVAARDLAPLLRQL   146
KO          AVINMNNGVLKVVRSKDAKTAAVPVAS-DAAPGIGDEVVTRVVPLTNVAARDLAPLLRQL   149
EC_H10407   AVVPMENDVLKVVKSSAAKVEPLPLVGEGSDNYAGDEMVTKVVPVRNVSVRELAPILRQM   165
VC          AVVEMDNGVLKVIKSKDAKTSAIPVLS-GEERANGDEVITQVVAVKNVSVRELSPLLRQL   146
            :*:   :   :::    .*.               *.: *::: : :  . :* *::* :

            N1 domain                                       N2 domain
            S7   S8   H4   S9   H5
PA          VPQYG--HLAAVPSANALIISDRSANIARIEDVIRQLDQKGSHDYSVINLRYGWVMDAAE   224
EC_K12      MDAGSVGNVVHYEPSNVLILTGRASTINKLIEVIKRVDVIGTEKQQIIHLEYASAEDLAE   207
KP          NDNAGAGSVVHYEPSNVLLMTGRAAVIKRLLTIVERVDNAGDRSVVTVPLSWASAAEVVK   206
KO          NDNAGAGSVVHYEPSNVLLMTGRAAVIKRLLTIVERVDNAGDRSVVTVPLAWASAADVVK   209
EC_H10407   IDSAGSGNVVNYDPSNVIMLTGRASVVERLTEVIQRVDHAGNRTEEVIPLDNASASEIAR   225
VC          IDNAGAGNVVHYDPANIILITGRAAVVNRLAEIIRRVDQAGDKEIEVVELNNASAAEMVR   206
                .   :.     :* ::::.*:: : ::  ::.::*  * .    : *  . . : ..

            N2 domain
            S10   S11   H6
PA          VLNNAMS----RGQAKGAAGAQVIADARTNRLIILGPPQARAKLVQLAQSLDTPTARSAN   280
EC_K12      ILNQLISESHGKSQMPALLSAKIVADKRTNSLIISGPEKARQRITSLLKSLDVEESEEGN   267
KP          LVTELN-KDTSKSALPGSMVANVVADERTNAVLVSGEPNSRQRIIAMIKQLDRQQAVQGN   265
KO          LVTELN-KDTSKSALPGSMVANVVADERTNAVLVSGEPNSRQRIIAMIKQLDRQQATQGN   268
EC_H10407   VLESLT-KNS-GENQPATLKSQIVADERTNSVIVSGDPATRDKMRRLIRRLDSEMERSGN   283
VC          IVEALN-KTTDAQNTPEFLKPKFVADERTNSILISGDPKVRERLKRLIKQLDVEMAAKGN   265
            ::                     :.:** *** ::: *    * ::  : : **     ..*

            N3 domain
            S12   H7   S13   S14
PA          TRVIRLRHNDAKTLAETLGQISEGMKNNGGQGGEQTGGGRPSNILIRADESTNALVLLAD   340
EC_K12      TRVYYLKYAKATNLVEVLTGVSEKLKDEKGNARKPSSSGAMDNVAITADEQTNSLVITAD   327
KP          TKVIYLKYAKAADLVEVLTGISSSLQSDKQSARPVA--AIDKNIIIKAHGQTNALIVTAA   323
KO          TKVIYLKYAKASDLVEVLTGISSTMQSEKQAAKPVA--ALDKNIIIKAHGQTNALIVTAA   326
EC_H10407   SQVFYLKYSKAEDLVDVLKQVSGTLTAAKEEAEGTVGSG-REIVSIAASKHSNALIVTAP   342
VC          NRVVYLKYAKAEDLVEVLKGVSENLQAEKGTGQPTT-SK-RNEVMIAAHADTNSLVLTAP   323
            .:*  *:: .*  *.:.*  :*   :       .          . : * *   :*:*:: *

            N3 domain         Secretin core domain
            H8   S15   S16
PA          PDTVNALEDIVRQLDVPRAQVLVEAAIVEISGDIQDAVGVQWAINKGGMGGTKTNFANTG   400
EC_K12      QSVQEKLATVIARLDIRRAQVLVEAIIVEVQDGNGLNLGVQWANKNVGAQ---QF-TNTG   383
KP          PDVMNDLERVIAQLDIRRPQVLVEAIIAEVQDADGLNLGIQWANKNAGMT---QF-TNSG   379
KO          PDVMNDLERVIAQLDIRRPQVLVEAIIAEVQDADGLNLGIQWANKNAGMT---QF-TNSG   382
EC_H10407   QDIMQSLQSVIEQLDIRRAQVHVEALIVEVAEGSNINFGVQWASKDAGLM---QFANGTQ   399
VC          QDIMNAMLEVIGQLDIRRAQVLIEALIVEMAEGDGINLGVQWGSLESGSV---IQYGNTG   380
             .  : :  :: :**: * ** :** *.*:       .*:**.  . *          .:
```

```
                 Secretin core domain
                 H9                                   H10      S17
PA          LSIGTLLQSLESNKAPES-------------------------------IPDGAIVGIGS   429
EC_K12      LPIFNAAQGVADYKKNGGI-------------------TSANPAWDMFSAYNGMAAGFFN   424
KP          LPISTAIAGANQYNKDGTI-------------------SSSL--ASALGSFNGIAAGFYQ   418
KO          LPISTAIAGANQYNKDGTV-------------------SSSL--ASALSSFNGIAAGFYQ   421
EC_H10407   IPIGTLGAAISQAKPQKGSTVISEN---GATTINPDTNGDLSTLAQLLSGFSGTAVGVVK   456
VC          ASIGNVMIGLEEAKDTTQTKAVYDTNNNFLRNETTTTKGDYTKLASALSSIQGAAVSIAM   440
             * .   .  . :                                      .*  ...

                 Secretin core domain
                 S18                 S19
PA          SSFGALVTALSANTKSNLLSTPSLLTLDNQKAEILVGQNVPFQTGSYTTNSEGSSNPFTT   489
EC_K12      GDWGVLLTALASNNKNDILATPSIVTLDNKLASFNVGQDVPVLSGSQTTS---GDNVFNT   481
KP          GNWAMLLTALSSSTKNDILATPSIVTLDNMQATFNVGQEVPVLTGSQTTS---GDNIFNT   475
KO          GNWAMLLTALSSSTKNDILATPSIVTLDNMEATFNVGQEVPVLTGSQTTS---GDNIFNT   478
EC_H10407   GDWMALVQAVKNDSSSNVLSTPSITTLDNQEAFFMVGQDVPVLTGSTVGS--NNSNPFNT   514
VC          GDWTALINAVSNDSSSNILSSPSITVMDNGEASFIVGEEVPVITGSTAGS--NNDNPFQT   498
            ..:  *: *:  ....::*::**: .:**  * : **::**. :** . .   ..* * *

                 Secretin core domain
                 S20                 S21                  S22
PA          VERKDIGVSLKVTPHINDGAALRLEIEQEISALLPNAQQR-NNTDLITSKRSIKSTILAE   548
EC_K12      VERKTVGTKLKVTPQVNEGDAVLLEIEQEVSSVDS---SSNSTLGPTFNTRTIQNAVLVK   538
KP          VERKTVGIKLKVKPQINEGDAVLLEIEQEVSSVADSASSTSSDLGATFNTRTVNNAVLVG   535
KO          VERKTVGIKLKVKPQINEGDSVLLEIEQEVSSVADAASSTSSDLGATFNTRTVNNAVLVG   538
EC_H10407   VERKKVGIMLKVTPQINEGNAVQMVIEQEVSKVEGQ-----TSLDVVFGERKLKTTVLAN   569
VC          VDRKEVGIKLKVVPQINEGNSVQLNIEQEVSNVLGA----NGAVDVRFAKRQLNTSVMVQ   554
            *:** :*   *** *::*:* :: : ****:* :           .     * ::.::.

                 Secretin core domain
                 S23            H11   H12      S24
PA          NGQVIVIGGLIQDDVSQAESKVPLLGDIPLLGRLFRSTKDTHTKRNLMVFLRPTVVRDSA   608
EC_K12      TGETVVLGGLLDDFSKEQVSKVPLLGDIPLVGQLFRYTSTERAKRNLMVFIRPTIIRDDD   598
KP          SGETVVVGGLLDKTVTDTADKVPLLGDIPVIGALFRSDSKKVSKRNLMLFIRPTIIRDRD   595
KO          SGETVVVGGLLDKSVSDTADKVPLLGDIPVIGALFRSTSKKVSKRNLMLFIRPTVIRDRD   598
EC_H10407   DGELIVLGGLMDDQAGESVAKVPLLGDIPLIGNLFKSTADKKEKRNLMVFIRPTILRDGM   629
VC          DGQMLVLGGLIDERALESESKVPLLGDIPLLGQLFRSTSSQVEKKNLMVFIKPTIIRDGV   614
             *: :*:***::.   :   *********::* **:       *:***:*::**::**

                 S domain
                 H13                  H14               H15
PA          GLAALSGKKYSDIRVIDGTRGPEGRP---------SILPTNANQLFDGQAVDLRELMTE-   658
EC_K12      VYRSLSKEKYTRYRQEQQQRIDGKSKALVGSEDL-PVL---DENTFNSHAPAPSSR----   650
KP          EYRQASSGQYTAFNNAQTKQRGKESSEASLSNDLLHIYPQQETQAFRQVSAAIDAFNLGG   655
KO          EYRQASSGQYTAFNDAQSKQRGKENNDAMLSNDLLEIYPRQDTAAFRQVSAAIDAFNLGG   658
EC_H10407   AADGVSQRKYNYMRAEQIYRDEQGL--SLMPHTAQPVLPAQNQ----ALPPEVRAFLNAG   683
VC          TADGITQRKYNYIRAEQLFRAEKGL--RLLDDASVPVLPKFGD--DRRHSPEIQAFIEQM   670
                 :  :*.  .  :  :                 :

PA          ----   658
EC_K12      ----   650
KP          RP--   657
KO          NL--   660
EC_H10407   RTR-   686
VC          EAKQ   674
```

XXX Signal peptide — β-sheet
---- No build — * Identical
Stabilising loop 7 — : High similarity
Helix — . Low similarity

Figure S3. PulD secondary structure assignment and sequence alignment. Aligned PulD sequences include *Pseudomonas aeruginosa* (PA, Uniprot code P35818), *Escherichia coli* K12

(EC_K12, P45758), *Klebsiella pneumoniae* (KP, A0A0E1CJT4), *Klebsiella oxytoca* (KO, A0A0H3H6N4), *Escherichia coli* H10407 (EC_H10407, E3PJ86), *Vibrio cholerae* (VC, P45779).

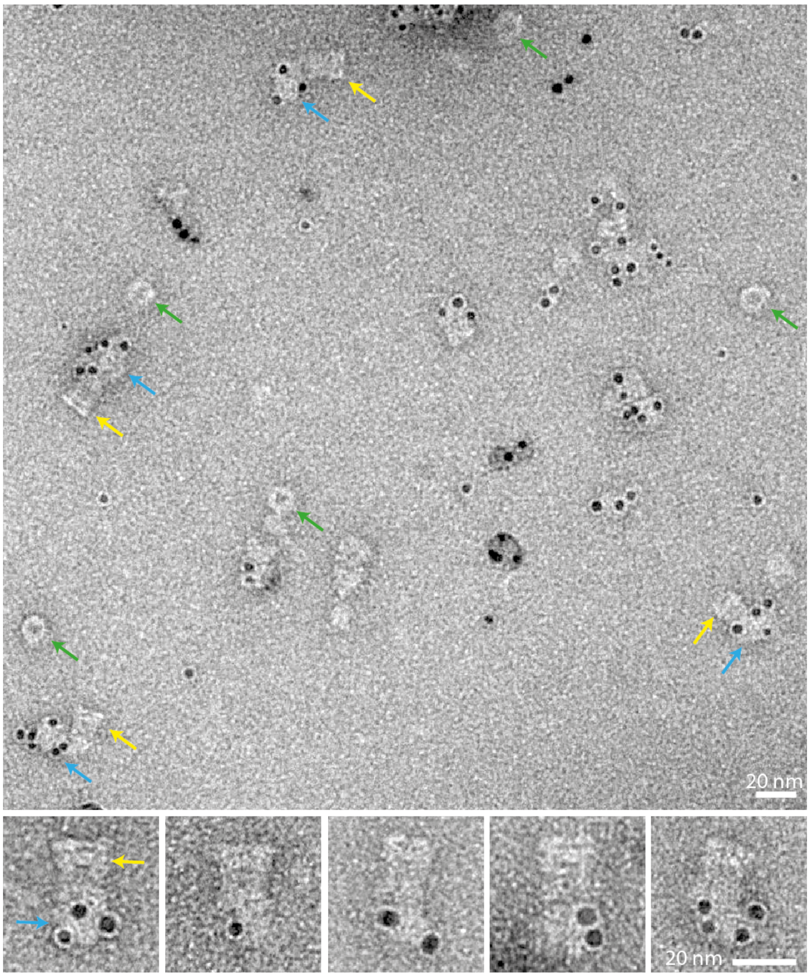

Figure S4. PulC N-terminus is located in the inner membrane assembly platform (AP). An overview negative stain EM image with a gallery of selected particles underneath. The PulC N-terminus was labelled with a hexahistidine tag at aa 61 within the $Pul_{CDELMNS}$ complex. Ni-NTA gold beads localize to the AP (blue arrow). Gold beads did not localize to the secretin when attached to the AP (side view, yellow arrow) or when dissociated from the AP (top view, green arrow).

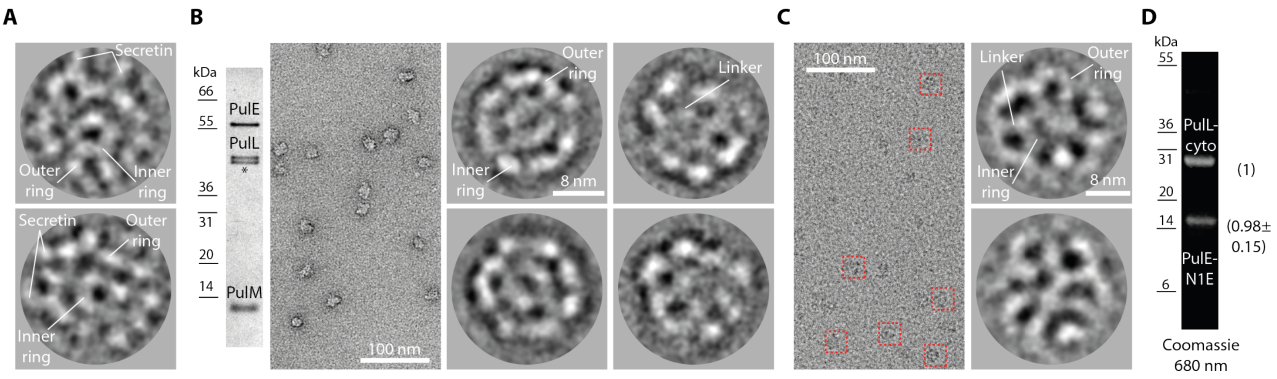

Figure S5. 2D EM analysis of the Pul$_{CDELMNS}$ assembly platform (AP) and the Pul$_{ELM}$ complex. (A) Gallery of cryo-EM 2D class averages of the Pul$_{CDELMNS}$ complex where the alignment and classification were focused on the AP only. A single preferred orientation was observed consistent with a bottom or end view. The base of the masked-out secretin is visible in the class averages. **(B)** GraFix purified Pul$_{ELM}$ complex and EM analysis. (Left) Silver stain SDS-PAGE gel showing purified Pul$_{ELM}$ complex. PulN bound weakly to the complex and was observed in only trace quantities after the GraFix ultracentrifugation step. (Right) Gallery of negative stain EM 2D class averages of the Pul$_{ELM}$ complex. The same single preferred orientation was observed as in **A**. **(C)** Gallery of cryo-EM 2D class averages of the Pul$_{ELM}$ complex. Note how the concentric ring ultrastructure is equivalent in both negative stain (as in **B**) and under cryo conditions. **(D)** SDS-PAGE of purified Pul$_{E\text{-}N1E/Lcyto}$ complex. Fluorescent emission Coomassie R250 stained gel imaged at 680 nm with associated stoichiometry and standard deviation in parentheses. Stoichiometry measurements were determined from two independent purifications.

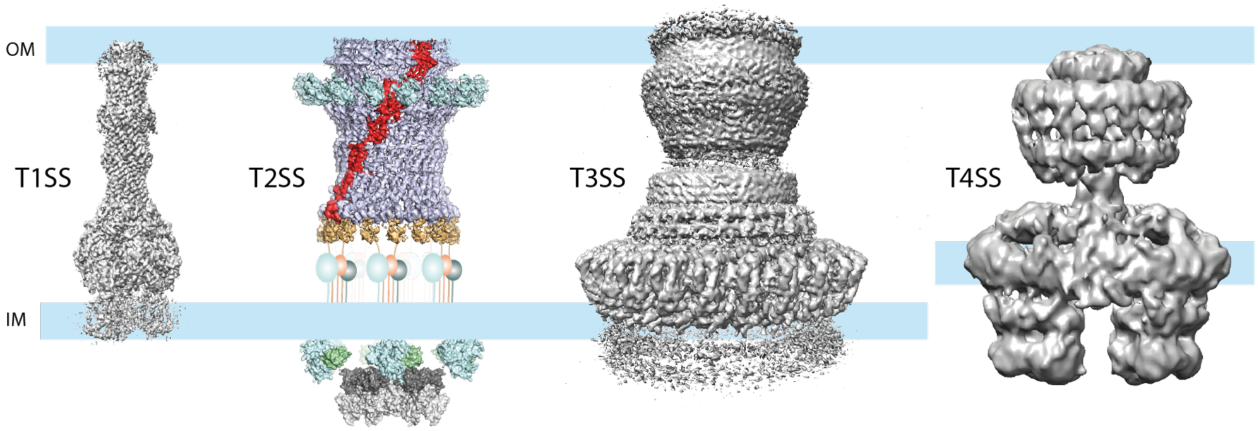

Figure S6. Comparison of bacterial type I-IV secretion systems. Substrate passes in an enclosed channel across the entire cell envelope in the type I (EMD 8636) and III (EMD 8400) secretion systems[3,4]. In the T2SS, the substrate must enter the periplasm before outer membrane translocation through the secretin channel. In the type IV secretion system[5], it is still unclear whether substrate passes across the cell envelope directly from the cytoplasm or is loaded first into the periplasm before being secreted.

Supplementary Table 1. 3D reconstruction and refinement statistics

**Data collection**		
Electron microscope	Titan Krios	
Voltage (kV)	300	
Pixel size (Å)	1.78	
Electron exposure ($e^-/Å^2$)	50	
Defocus range (µm)	2-4.5	
Images	3427	
**3D reconstruction**		
Final particles	7284	
Resolution (Å)	4.3	
FSC threshold	0.143	
B factor ($Å^2$)	−142	
**Refinement**		
Model	PulD*	PulD*-PulS-PulC
Homology model template		PulC HR- PDB 3OSS<br>PulS- PDB 4K0U
**Model composition**		
Total atoms	64920	85170
Total residues	8655	11295
**R.m.s. deviations**		
Bond length (Å)	0.011	0.011
Angles (°)	1.4	1.3
**Validation**		
MolProbity score	2.66	2.58
Clashscore	38.01	36.54
Poor rotamers (%)	0	0
**Ramachandran plot**		
Favored (%)	88.07	90.01
Outliers (%)	0	0
**B-factors/ADPs**		
Minimum	36.5	36.5
Maximum	294.9	425.3
Mean	103.7	161.5
**Real-space correlation**		
Masked map	0.78	0.73
Unmasked map	0.76	0.71

*identical models

Supplementary Table 2. Cloning primers

Primer pair 1	taacgagggcaaaaaatgccagtttctgtgatgaggttaacaaata<br>aggtcaagcttattactagaccatctgctgccagagaaaaata
Primer pair 2<br>(pASK3c)	taataagcttgacctgtgaagtg<br>tttttgccctcgttatctagatttttg
Primer pair 3	ccacccgcagttcgaaaaataagatggccctgtttcgctatcaggcg<br>tcgaactgcgggtggctccagacgctttccgtcgtttgctgttcg
Primer pair 4	aggagatataccatgattcattattttgattactctatgatg<br>tgttcgacttaagcattagtcgctgcgcaagctggc
Primer pair 5<br>(pCDF-duet)	tgcttaagtcgaacagaaagtaatc<br>catggtatatctccttattaaag
Primer pair 6	actacaaagacgatgacgacaagtaatgcttaagtcgaacagaaagtaat<br>catcgtctttgtagtcggatccgtcgctgcgcaagctggcaata
Primer pair 7	taacgagggcaaaaaatgacgccagccgccgaacgccgt<br>gtggctccaagcgctgacgctttccgtcgtttgctgttcggtg
Primer pair 8<br>(pASK3c)	agcgcttggagccacccgcagttc<br>tttttgccctcgttatctagatttttg
Primer pair 9	aggagatataccatgaacaagattaatgcttctccccaggcc<br>tttgtagtcggatcctttttgctcctgtggtaaccattttccctg
Primer pair 10<br>(pCDF-duet/flag tag)	ggatccgactacaaagacgat<br>catggtatatctccttattaaag
Primer pair 11	taacgagggcaaaaaatgacgccagccgccgaacgccgtcc<br>gtggctccaagcgctgctgtcgagcagatcgtcggtgtccg
Primer pair 12	aggagatataccatgaacaagattaatgcttctccccaggcc<br>tttgtagtcggatccggcgaactccccctgcagcagactga
Primer pair 13	catcaccatcatcaccactaattgccaaccatgaaaagcctgcgaaaaat<br>gtgatgatggtgatggccttcatcccccagcgcgaaagcaatatcgtgtc

Supplementary References

1. Kucukelbir, A., Sigworth, F.J. & Tagare, H.D. Quantifying the local resolution of cryo-EM density maps. *Nat Methods* **11**, 63-5 (2014).
2. Jakobi, A.J., Wilmanns, M. & Sachse, C. Model-based local density sharpening of cryo-EM maps. *Elife* **6**, e27131 (2017).
3. Wang, Z. et al. An allosteric transport mechanism for the AcrAB-TolC multidrug efflux pump. *Elife* **6**, e24905 (2017).
4. Worrall, L.J. et al. Near-atomic-resolution cryo-EM analysis of the *Salmonella* T3S injectisome basal body. *Nature* **540**, 597-601 (2016).
5. Low, H.H. et al. Structure of a type IV secretion system. *Nature* **508**, 550-553 (2014).
