# Supplementary material for: Core architecture of a bacterial type II secretion system
Source: Nat Commun. 2019 Nov 28;10:5437. doi: 10.1038/s41467-019-13301-3 (PMC6882859; doi:10.1038/s41467-019-13301-3)
Supplement: Supplementary file 3 — Source Data [file 41467_2019_13301_MOESM3_ESM.pdf]

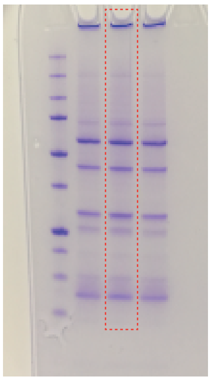

Purification of the Pul$_{CDELMNS}$ complex. Coomassie R250 stained gel without phenol treatment. Dotted rectangle indicates relevant SDS-PAGE lane. See Figure 1B.

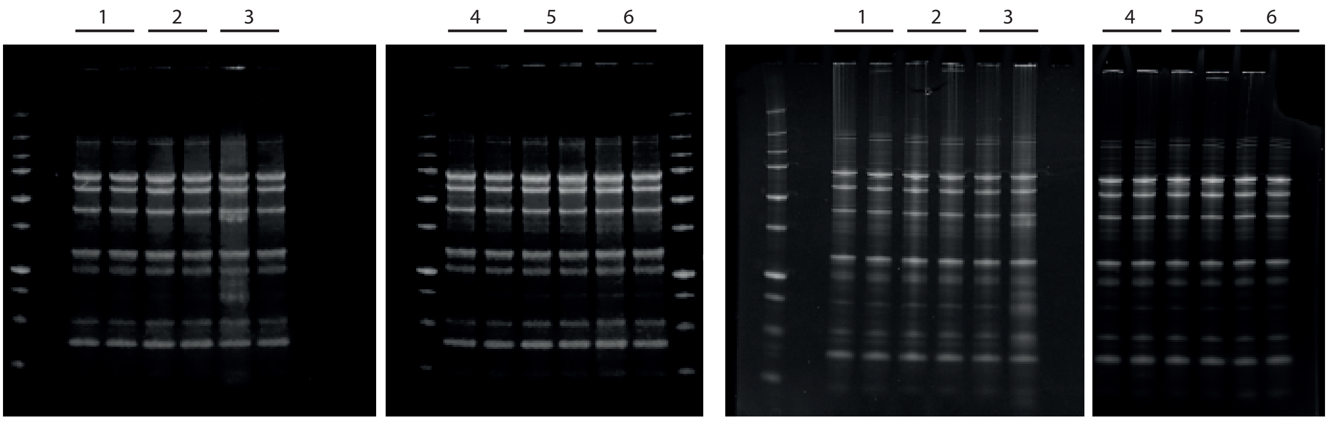

Stoichiometry of the Pul$_{CDELMNS}$ complex. Fluorescent emission Coomassie R250 stained gels imaged at 680 nm with phenol extraction (left). Fluorescent emission Sypro Ruby stained gel imaged at 302 nm with phenol extraction (right). 6 independent purifications were undertaken with duplicate samples run. See Figure 1B.
